# *Architrypethelium murisporum* (Ascomycota, Trypetheliaceae), a remarkable new lichen species from Thailand challenging ascospore septation as an indicator of phylogenetic relationships

**DOI:** 10.3897/mycokeys.25.23836

**Published:** 2018-05-10

**Authors:** Theerapat Luangsuphabool, H. Thorsten Lumbsch, Jittra Piapukiew, Ek Sangvichien

**Affiliations:** 1 Department of Biology, Faculty of Science, Ramkhamhaeng University, Bangkok, Thailand; 2 Science & Education, The Field Museum, Chicago, Illinois, USA; 3 Department of Botany, Faculty of Science, Chulalongkorn University, Bangkok, Thailand

**Keywords:** Lichens, taxonomy, phylogeny, tropical diversity, Southeast Asia, Trypetheliales

## Abstract

*Architrypethelium murisporum* Luangsuphabool, Lumbsch & Sangvichien is described for a crustose lichen occurring in dry evergreen forest in Thailand. It is characterised by a green to yellow-green corticated thallus, perithecia fused in black pseudostromata with white rim surrounding the ostiole and small, hyaline and muriform ascospores. Currently, all species in the genus *Architrypethelium* have transversely septate ascospores, hence the discovery of this new species indicates that ascospore septation is variable within the genus, similar to numerous other groups of lichen-forming ascomycetes. Phylogenetic analyses of two loci (mtSSU and nuLSU) supported the position of the new species within *Architrypethelium*. This is the first report of the genus in Southeast Asia.

## Introduction

The genus *Architrypethelium* Aptroot (Ascomycota, Dothideomycetes, Trypetheliales) includes crustose lichens with perithecioid ascomata growing on tree bark in the tropics (Aptroot 1991, Aptroot et al. 2008, Aptroot and Lücking 2016). The genus accommodates species with a corticate thallus, solitary or aggregate ascomata with apical or eccentric ostioles, a clear or inspersed hymenium and hyaline or brown, 3–5 septate, transversely septate ascospores (Aptroot et al. 2008, Aptroot and Lücking 2016). Although, *Architrypethelium* is morphologically similar to *Astrothelium* species, the two genera have been shown to be distantly related. The latter genus fell into two clades (Lücking et al. 2016) with one being a sister group to *Architrypethelium*. Phenotypically *Architrypethelium* differs from *Astrothelium* in having predominantly large ascospore without diamond-shaped lumina when mature (Aptroot 1991, Aptroot et al. 2008, Nelsen et al. 2014, Aptroot and Lücking 2016, Lücking et al. 2016b). Another genus with muriform ascospores is *Aptrootia*, which also shares an astrothelioid stage in the young ascospores (Lücking et al. 2016) and the genus formed a sistergroup to a clade including *Architrypethelium* and *Astrothelium* p.pt. further calling the generic delimitation in the family in question. Morphologically, *Aptrootia* differs from *Astrothelium* in having dark brown ascospores with a hard outer shell (Lücking et al. 2016). While most genera in Trypetheliaceae, such as *Astrothelium* s.str., *Bathelium*, *Polymeridium* and *Viridothelium* include species with various ascospore types (Hyde et al. 2013, Nelsen et al. 2014, Aptroot and Lücking 2016, Lücking et al. 2016b), the species of *Architrypethelium* shared a similar ascospore morphology (Nelsen et al. 2014, Lücking et al. 2016b).

Previously, three species were accepted in *Architrypethelium* (Aptroot 1991, Aptroot et al. 2008). Recently, the numbers of species increased with the description of two new species and two combinations into the genus (Aptroot and Lücking 2016, Flakus et al. 2016, Lücking et al. 2016a). Currently, seven species are accepted in *Architrypethelium*, viz. *Architrypethelium columbianum* (Nyl.) Aptroot & Lücking, *Architrypethelium grande* (Kremp.) Aptroot & Lücking, *Architrypethelium hyalinum* Aptroot, *Architrypethelium lauropaluanum* Lücking, Nelsen & Marcelli, *Architrypethelium nitens* (Fée) Aptroot, *Architrypethelium penuriixanthum* Flakus & Aptroot, and *Architrypethelium uberinum* (Fée) Aptroot (Aptroot 1991, Aptroot et al. 2008, Aptroot and Lücking 2016, Flakus et al. 2016, Lücking et al. 2016a). All species are known from the Neotropics, except *A. uberinum*, which is also known from Oceania (Aptroot and Lücking 2016, Flakus et al. 2016, Lücking et al. 2016a), suggesting a pantropical distribution (Aptroot and Lücking 2016). Until now, the genus *Architrypethelium* has not been known from southeast Asia. Here we describe a new species from Thailand, which has a rich pyrenocarpous lichen flora (Buaruang et al. 2017), with muriform ascospores, confirming its presence in southeast Asia. Further, we provide phylogenetic evidence to support its placement in the genus *Architrypethelium* and hence demonstrating that the ascospore septation is also variable in this genus.

## Material and methods

### Specimen collection and phenotypical studies

The material of the new species was found in a dry evergreen forest of the north-eastern region in Thailand. Morphology was studied using an Olympus SZ11 dissecting microscope and free hand sections were mounted in distilled water and studied using an Olympus BX53 compound microscope with differential interference contrast (DIC) (Olympus U-DICT), connected to a Canon EOS650 digital camera. Secondary metabolites were studied using thin-layer chromatography (TLC) with standard solvent A (Orange et al. 2001, Lumbsch 2002).

### Molecular data

Genomic DNA of the holotype was extracted from the dried lichen thallus using the CTAB method with chloroform precipitation (Cubero and Crespo 2002). DNA amplification was performed for mitochondrial small subunit ribosomal DNA (mtSSU) and nuclear large subunit ribosomal DNA (nuLSU) using primer pairs mrSSU1 (Zoller et al. 1999) with MSU7 (Zhou and Stanosz 2001) and LR0R with LR3 (Vilgalys and Hester 1990), respectively. PCR reaction mixture was prepared in a total volume of 50 μl, consisting of 5 μl of 10× Pfu Buffer with MgSO_4_, 2mM of dNTP mix, 20 μM of each primer, 1·25 U of *Pfu* DNA Polymerase (Thermo Fisher Scientific Inc.) and 5 μl of 1/10 dilution of DNA solution. PCR was performed using a thermal cycler Life ECO (Hangzhou Bioer Technology Co., China) as follows: initial denaturation for 1 min at 94 °C and 38 cycles of 94 °C for 1 min, 52 °C for 45 s (LR0R/LR3) and 53 °C for 45 s (mrSSU1/MSU7), followed by an extension at 72 °C for 1 min and a final extension at 72 °C for 5 min. DNA purification and sequencing methods followed Luangsuphabool et al. (2016).

### Phylogenetic analysis

The new sequences were aligned with other species of *Architrypethelium* and other Trypetheliaceae from GenBank (Table 1). *Aptrootia* and *Astrothelium* s. lat. have been shown to be the sister groups to *Architrypethelium* (Lücking et al. 2016b) and two taxa of *Bathelium madreporiforme* were used as the outgroup. The DNA datasets (mtSSU and nuLSU) were aligned separately using MUSCLE (Edgar 2004) and improved manually using MEGA v.7 (Kumar et al. 2016). The nucleotide substitution model for maximum likelihood (ML) and Bayesian inference (BI) analyses was chosen using jModelTest v.2.1.4 (Darriba et al. 2012) with the Akaike Information Criterion (AIC). The ML tree was performed on the CIPRES supercomputer using the programme RAxML-HPC2 v.8.2.10 on XSEDE (Miller et al. 2010) and bootstrap values were estimated with 1000 pseudo-replicates. Bayesian inference analysis and posterior probabilities were calculated using MrBayes v.3.2.1 (Ronquist and Huelsenbeck 2003) with the Markov chain Monte Carlo (MCMC) algorithm. Four chains and two independent runs were carried out with 10 million generations. Every 100th tree was saved into a file and aborting the analysis was set at the mean standard deviation < 0·01. Tree topology of both ML and BI analyses was illustrated using FigTree v.1.4.2 (http://tree.bio.ed.ac.uk/software/figtree/).

**Table 1.** Species, location, voucher information and GenBank accession numbers for samples used in this study. Newly obtained sequences in bold and missing data are indicated by [–].

Species	Isolate	Country	Voucher information	GenBank accession No.	
				mtSSU	nuLSU
*Aptrootia elatior*	MPN560B	New Zealand	*Knight* O61815 (OTA)	KM453821	KM453754
*A. robusta*	MPN235B	Australia	*Lumbsch* 20012 (F)	KM453822	KM453755
*A. terricola*	DNA1501	Costa Rica	*Lücking* 17211 (F)	DQ328995	KM453756
*Architrypethelium lauropaluanum*	MPN48	Peru	*Nelsen* Cit1P (F)	KX215566	KX215605
*A. nitens*	MPN257	Panama	*Lücking* 27038 (F)	KM453823	KM453757
*A. uberinum*	MPN489	Brazil	*Nelsen* s. n. (F)	[–]	KM453758
*A. murisporum*	UBN215	Thailand	*Luangsuphabool* 031332 (RAMK)	**LC361339**	**LC361340**
*Astrothelium endochryseum*	MPN436	Brazil	*Lücking* 31088 (F)	KM453837	KM453772
*A. subendochryseum*	MPN202B	El Salvador	*Lücking* 28121 (F)	[–]	KX215659
*A. scorizum*	MPN336	Brazil	*Lücking* 29814 (F)	KM453872	KM453808
*A. obtectum*	MPN422	Brazil	*Lücking* 31242 (F)	KM453832	KM453767
*A. laevithallinum*	MPN442	Brazil	*Lücking* 31061 (F)	KM453836	KM453771
*A. subinterjectum*	MPN157	Brazil	*Nelsen* B15 (F)	KX215583	KX215660
*Bathelium madreporiforme*	NAN95	Thailand	*Luangsuphabool* 027903 (RAMK)	LC128029	LC127414
*B. madreporiforme*	UBN147	Thailand	*Luangsuphabool* 027904 (RAMK)	LC128028	LC127413

## Results and discussion

**Figure 1.**
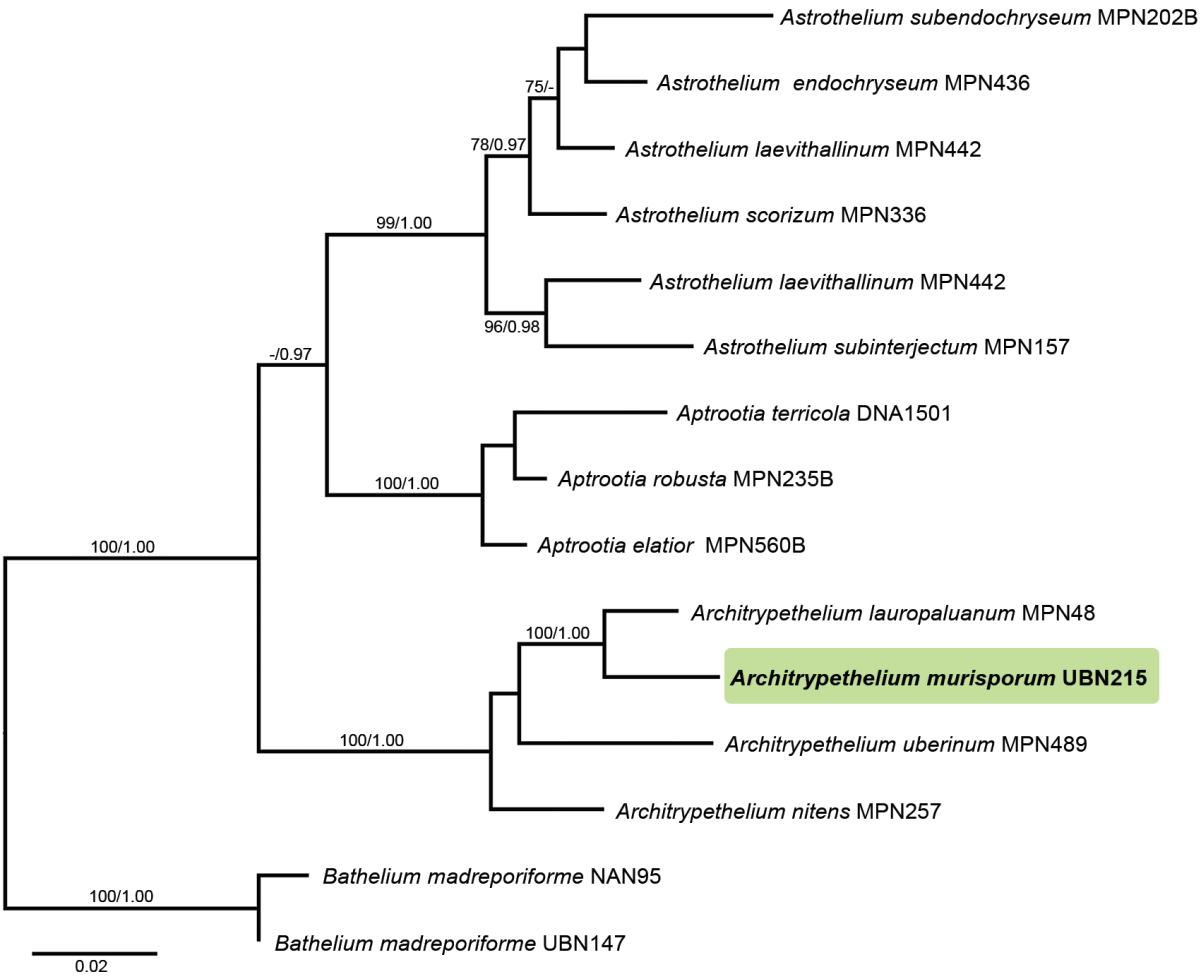
Phylogenetic relationships of *Architrypethelium* and sister genera based on a combined data set of two DNA loci (mtSSU and nuLSU rDNA). Bootstrap values ≥ 70% and posterior probabilities ≥ 0.95 are shown at above and below branches.

Two new DNA sequences of mtSSU and nuLSU were generated for this study (Table 1). The alignment matrix contained 609 unambiguously aligned nucleotide position characters, including 200 mtSSU and 409 nuLSU positions. The GTR+I+G model was chosen as the best-fit model for phylogenetic analyses. The topology of single locus analyses did not show any conflicts and hence the combined data set was used for the analysis. The posterior probabilities of the BI analysis together with the ML bootstrap values are both shown in the ML tree (Fig. 1).

**Figure 2.**
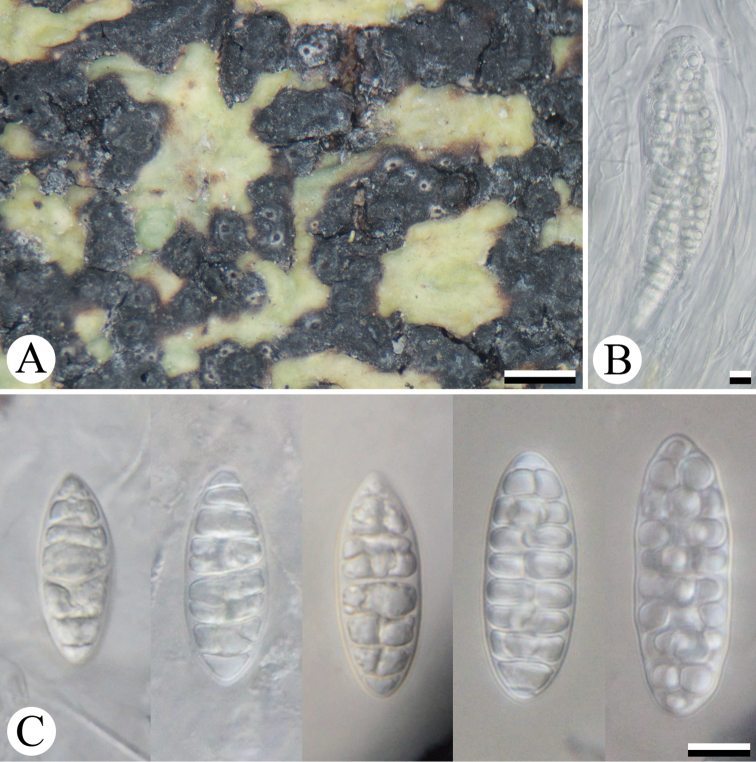
Morphological characters of *Architrypethelium murisporum* (holotype): **A** thallus and pseudostromata with ascomata **B** ascus with ascospores and **C** ascospores. Scale bars: 1 mm (**A**); 10 μm (**B–C**).

The tree topology supported the fact that the new species is part of the genus *Architrypethelium* with strong support values (Fig. 1). Although the morphological characters of the new species would place it in the genus *Astrothelium* (Fig. 2), the shape of ascospore lumina is somewhat different from *Astrothelium* in having rounded-shaped lumina (Fig. 2C) (Aptroot and Lücking 2016). This new species seems to be closer related to species with hyaline ascospore (*Architrypethelium lauropaluanum*) than brown ascospores (*A. nitens* and *A. uberinum*) (Aptroot 1991, Aptroot et al. 2008, Lücking et al. 2016a). So far, all species in *Architrypethelium* had large, transversely septate ascospores (Aptroot and Lücking 2016). However, our new species has small, muriform ascospores (Fig. 2B–C). The ascospore ontogeny in the new species resembles that of *Architrypethelium* spp. (Sweetwood et al. 2012), but continues septation to form muriform spores and the endospore is reduced when mature.

The variation of ascospore size and septation in *Architrypethelium* is not surprising given the variation of ascospores in other genera of Trypetheliaceae. This phenomenon is also commonly found in many genera in families of non-lichenised ascomycetes, viz. Lophiostomataceae and Melanommataceae (Mugambi and Huhndorf 2009) and lichenised families, such as Graphidaceae and Pyrenulaceae (Lücking 2009, Aptroot 2012, Weerakoon et al. 2012, Aptroot and Lücking 2016, Gueidan et al. 2016), which supports the fact that ascospore characters are often poor predictors of phylogenetic relationships (Nelsen et al. 2014, Lücking et al. 2016b).

## Taxonomic treatment

### Architrypethelium murisporum

Taxon classificationFungiTrypethelialesTrypetheliaceae

Luangsuphabool, Lumbsch & Sangvichien sp. nov.

MB823970

Figure 2

#### Type.

THAILAND. Ubon Ratchathani Province: Na Pho Klang, Khong Chiam District, 15°31'N, 105°35'E, ca. 130 m alt., dry evergreen forest, on tree bark, 27 November 2012, *T. Luangsuphabool* RAMK 031332 (holotype: RAMK).

#### Diagnosis.

Characterised within the genus by having small, hyaline and muriform ascospores.

#### Etymology.

The specific epithet refers to the muriform ascospore character of the new species.

#### Description.

Thallus crustose, corticate, thick, green to yellow-green, smooth to uneven, with cortex 40–125 μm thick, medulla 20–75 μm thick, prothallus black. Algae trentepohlioid, cells 18–65 μm wide. Ascomata perithecia, pyriform, black, 0.45–0.60 mm diam., erupent to prominent, fused into a pseudostroma, not covered by thallus. Ascoma wall carbonised, up to ca. 145 μm thick. Ostiole apical, black, not shared, with a white annulus surrounding the ostiolar region. Pseudostroma forming raised black lines, irregular in shape or forming a partial network on the thallus. Hamathecium hyaline, not inspersed with droplets or granules, consisting of branched and anastomosing paraphyses, 1.5–2.5 µm thick. Asci clavate to cylindrical, 150–200 × 32–50 µm. Ascospores 8 per ascus, hyaline, muriform with 6–9 transverse and 1–2 longitudinal septa per tier near centre of spore in optical section, narrowly ellipsoid, 35–50 × 13–15.5 μm. Pycnidia not observed.

#### Secondary chemistry.

Thallus UV–, K–, C–, KC–, PD– ; pseudostroma UV–, K–, C–, KC–, PD– . TLC: no substances detected.

#### Distribution and ecology.

The new species was found in north-eastern Thailand, growing in a dry evergreen forest on tree bark. It is only known from the type locality.

#### Notes.

*Architrypethelium murisporum* is morphologically similar to *Astrothelium keralense* (Upreti & Ajay Singh) Aptroot & Lücking and *A. variatum* (Nyl.) Aptroot & Lücking in having hyaline, small and muriform ascospores, but differs in having ascomata fused into a pseudostroma and not covered by the thallus (ascomata solitary, covered by the thallus in *A. keralense* and ascomata covered by thallus except ostiole regions in *A. variatum*), narrowly ellipsoid ascospores (fusiform in both *Astrothelium* spp.). Also the ascospore size (35–50 × 13–15.5 μm) differs from *A. keralense* (50–60 x 15–20 μm) and *A. variatum* (24–35 x 11–13 μm). The placement of the new species in *Architrypethelium* is supported by molecular evidence (Fig. 1), but it is unlikely to be confused with any of the currently accepted species in that genus due to the differences in ascospore size and septation (Aptroot et al. 2008, Aptroot and Lücking 2016, Flakus et al. 2016, Lücking et al. 2016a). The new taxon has muriform and relatively small ascospores (ca. 50 µm, long) (Fig. 2), whereas other *Architrypethelium* species have transversely septate ascospores (3–5 septate), that are longer than 90 µm (Aptroot 1991, Aptroot et al. 2008, Aptroot and Lücking 2016, Flakus et al. 2016, Lücking et al. 2016a).

## Supplementary Material

XML Treatment for Architrypethelium murisporum
